# Effects of Ursodeoxycholic Acid Treatment for Intrahepatic Cholestasis of Pregnancy on Maternal and Fetal Outcomes

**DOI:** 10.7759/cureus.70800

**Published:** 2024-10-03

**Authors:** Madiha Iqbal, Zia Muhammad, Naheed Akhter, Samina Shams Alam

**Affiliations:** 1 Department of Obstetrics and Gynecology, Hayatabad Medical Complex Peshawar, Peshawar, PAK; 2 Department of Pediatrics, Khyber Teaching Hospital, Peshawar, PAK; 3 Department of Obstetrics and Gynecology, Khyber Teaching Hospital, Peshawar, PAK; 4 Department of Neonatology, Hayatabad Medical Complex Peshawar, Peshawar, PAK

**Keywords:** fetal, icp, maternal, serum bile acid, udca

## Abstract

Background: Intrahepatic cholestasis of pregnancy (ICP) appears in the second or third trimester of pregnancy and is characterized by pruritus and elevated blood bile acid (BA) levels. Complications from these symptoms may include preterm birth, fetal distress, or stillbirth. Although the precise causes of ICP are unknown, genetic, hormonal, and environmental variables may be involved. First-line treatment for ICP is ursodeoxycholic acid (UDCA), which improves bile flow and consequently lowers BA levels and pruritus.

Objective: The objective of this study is to investigate the impact of UDCA therapy on maternal and fetal outcomes in women with ICP.

Materials and methods: This was a prospective observational study of 123 pregnant women with ICP, aged between 20 and 45 years who were diagnosed clinically (pruritus) supported by abnormal laboratory results including elevated serum BA levels, and abnormalities in liver function tests, over the course of three years, from July 2021 to June 2024. Every patient received UDCA, commencing at 10-15 mg/kg/day and being titrated according to clinical guidelines. Maternal and fetal outcomes were tracked for the duration of the pregnancy, with data being collected at baseline (15 ± 1 weeks) and every two weeks until delivery.

Results: The mean age of the study participants was 29.6 ± 5.4 years, with the youngest patient being 20 years and the oldest being 45 years. Most women were multipara 65.9%, and the mean BMI was 27.8 ± 3.5 kg/m ². The mean time of gestational age at ICP diagnosis was 31.2 ± 2.7 weeks, and the time of gestational age at delivery was 37.1 ± 2.4 weeks. On average, the serum BA level at diagnosis was 23.5 ± 8.1 µmol/L.

Conclusion: In the majority of ICP patients with good fetal outcomes, UDCA not only normalizes serum BA levels but also reduces maternal symptoms. In addition to addressing patient response variability to this therapy and optimizing dissemination procedures, the researchers expect that the results of this study will support the continued use of UDCA as first-line treatment for ICP, at least until more evidence becomes available.

## Introduction

Intrahepatic cholestasis of pregnancy (ICP) is a rare liver disorder that manifests in the second part of pregnancy and is characterized by pruritus, elevated serum bile acid (BA) levels, and occasionally liver failure [1]. Preterm, fetal discomfort, and, in the worst cases, stillbirth are among the serious outcomes that the condition may cause for both the mother and the fetus [2]. The exact cause of ICP is still unidentified but it is known to be complex and impacted by environmental, hormonal, and genetic factors [3]. It is believed that the pregnancy hormones progesterone and estrogen interfere with normal bile flow by reducing the liver’s ability to excrete BAs. As a result, BAs accumulate in the mother’s bloodstream, so the developing fetus cannot effectively process and eliminate these acids.

Ursodeoxycholic acid (UDCA) is the most used treatment for ICP [4]. UDCA, the hydrophilic BA molecule, is believed to lower BA tiers, enhance bile flow, and protect against symptoms associated with toxicity. While the benefits for mothers are obvious, UDCA is also thought to have a number of positive impacts on the fetus, such as a decreased chance of preterm birth, meconium-stained amniotic fluid, and fetal distress [5,6].

It seems that the mechanism of action of UDCA is to remove hydrophobic, poisonous BAs from the BA pool [7]. Despite being a commonly used medication in clinical practice, there are still differences of opinion about UDCA. Some studies suggest that UDCA is an effective treatment, improving several clinical outcomes, such as reducing serum BA levels and relieving pruritus in patients with ICP [8,9]. However, other studies have reported that UDCA has no significant effect on certain parameters, such as maternal or fetal circulation and BA clearance [10,11].

Additional research is required to determine how UDCA affects the evolution of maternal and fetal outcomes, given the previously observed issues and inconsistent results. This study aims to evaluate the anti-ICP impact of UDCA while taking fetal and maternal outcomes into account.

## Materials and methods

This study was a prospective observational study, carried out at Gynae B unit Hayatabad Medical Complex & Peads “A” Unit Khyber Teaching Hospital Peshawar, over the course of three years, from July 2021 to June 2024. A total of 123 pregnant women diagnosed with ICP were enrolled.

ICP was diagnosed when pregnant women presented with pruritus without a rash, primarily in the third trimester, along with elevated serum BAs (>10 µmol/L), and abnormal liver function tests (LFTs), particularly elevated alanine aminotransferase (ALT) and/or aspartate aminotransferase (AST). The diagnostic threshold for serum BAs and LFTs was consistent with guidelines from the Royal College of Obstetricians and Gynaecologists (RCOG)

Pregnant women aged 20-45 years, those with ICP diagnosed based on clinical symptoms like pruritus and laboratory investigations such as elevated serum BA (>10 µmol/L) and abnormal LFTs, such as increased levels of ALT and AST, and singleton pregnancy cases were included in the study. Women with known liver disease or other cholestatic diseases, multiple pregnancies, known fetal anomalies or genetic disorders, UDCA allergy, any contraindications to UDCA, hepatitis ABC, or known hematological disorder patients were excluded from the study.

The study protocol was approved by the institutional review board of Hayatabad Medical Complex Peshawar under reference# 2290. Written informed consent was obtained from all patients before the commencement of treatment after detailing the study’s objectives, benefits, and associated risks. UDCA (10-15 mg/kg/day) was administered orally to all of our qualified patients in divided doses of one to three times. The pill number and a follow-up patient interview were used to track it.

Maternal data comprised age, parity, body mass index, measurements of recreation, clinical symptoms, and pruritus severity assessed using a visual analog scale; liver functional tests, gestational age at symptoms and distributions, reactions, maternal complications; gestational diabetes and pre-eclampsia. Fetal data comprised the number of biweekly ultrasound assessments, fetal heart rate (FHR) monitoring, amniotic liquid score, and meconium staining, natal tone, the duration of birth, the availability of information on the distribution of caesarian sections and inductions, and neonatal intensive care unit (NICU).

Data were analyzed using IBM SPSS Statistics for Windows, Version 25 (Released 2017; IBM Corp., Armonk, New York, United States). Maternal and fetal outcomes were compared between patients who achieved normal versus non-normalized serum BA levels through the chi-square test for categorical variables and the independent t-test for continuous variables. The difference was considered statistically significant when the p-value was <0.05.

## Results

A total of 123 pregnant women with ICP were included in the study. The mean age of the study participants was 29.6 ± 5.4 years, with the youngest patient being 20 years and the oldest being 45 years. Most women were multipara 81(65.9%), the average BMI was 27.8 ± 3.5 kg/m ². The mean time of gestational age at ICP diagnosis was 31.2 ± 2.7 weeks, and the time of gestational age (GA) at delivery was 37.1 ± 2.4 weeks. On average, the serum BA level at diagnosis was 23.5 ± 8.1 µmol/L (Table 1).

**Table 1:** Baseline characteristics of the study population

Characteristic	Value
Age (years), mean ± SD	29.6 ± 5.4
Age range (years)	20 - 45
Multiparous (%)	81(65.9%)
Body mass index (BMI) (kg/m²), mean ± SD	27.8 ± 3.5
Gestational age at diagnosis (weeks), mean ± SD	31.2 ± 2.7
Gestational age at delivery (weeks), mean ± SD	37.1 ± 2.4
Serum bile acid level at diagnosis (µmol/L), mean ± SD	23.5 ± 8.1

**Figure 1:**
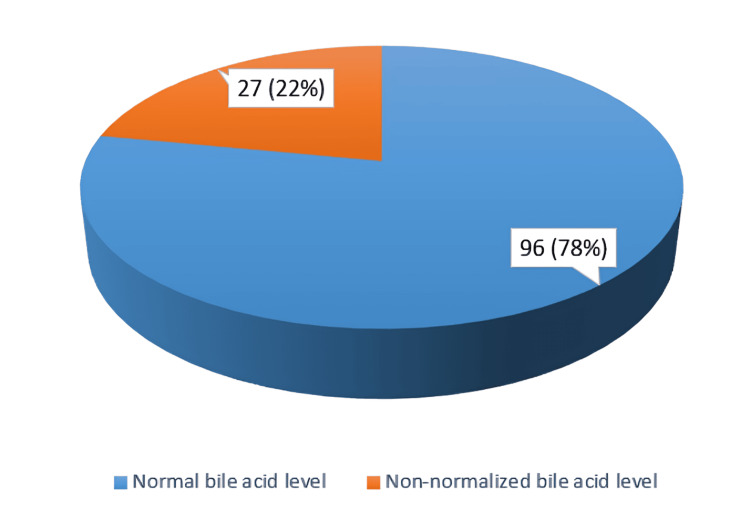
Normal and non-normalized bile acid levels

Among the total cases, 96 (78%) had normalized the BA levels that were <10 µmol/L by the time of delivery (Figure 1).

The application of UDCA treatment led to a significant reduction in pruritus severity. At the baseline, the average score on the VAS was 7.3 ± 1.5. Three weeks after the treatment, the score had reduced to 3.8 ± 1.1, ideally implying considerable relief. Four weeks later, the mean VAS was reduced to 1.8 ± 1.0, which was a significant relief by the time of delivery. UDCA was significantly effective in reducing the serum BA levels. By the time of the fourth week, there was a decrease in BA levels from 23.5 ± 8.1 µmol/L at diagnosis to 11.4 ± 4.3 µmol/L. LFTs greatly improved after the application of UDCA. The mean ALT decreased from 65.7 ± 18.4 U/L at diagnosis to 32.6 ± 11.3 U/L by the day of delivery. Similarly, the AST levels decreased from 59.2 ± 16.9 U/L to 28.7 ± 9.8 U/L. ALP also decreased from 220.5 ± 45.8 U/L to 167.3 ± 33.6 U/L. Regarding maternal complications, the incidence of gestational diabetes was found in 9(7.3%) of the cases and preeclampsia in 5(4%). No cases of severe liver dysfunction or UDCA-related adverse effects were reported during the study (Table 2).

**Table 2:** Maternal outcome

Outcome	Baseline Value	Post-Treatment Value	p-Value
Pruritus severity (VAS score), mean ± SD	7.3 ± 1.5	1.8 ± 0.7 (at delivery)	0.061
Serum bile acid levels (µmol/L), mean ± SD	23.5 ± 8.1	11.4 ± 4.3 (at 4 weeks)	0.05
ALT (U/L), mean ± SD	65.7 ± 18.4	32.6 ± 11.3 (at delivery)	0.043
AST (U/L), mean ± SD	59.2 ± 16.9	28.7 ± 9.8 (at delivery)	0.001
ALP (U/L), mean ± SD	220.5 ± 45.8	167.3 ± 33.6 (at delivery)	0.051
Gestational diabetes (%)	N/A	9(7.3%)	-
Pre-eclampsia (%)	N/A	5(4%)	-

Maternal FHR monitoring was within normal limits in almost all cases. However, an abnormal pattern consistent with the fetus’s distress was noted in 15 (12.2%) of cases. OUS showed normal amniotic fluid index in 89.4% of cases and oligohydramnios in 13(10.6%). The meconium-stained amniotic fluid was present in 18(14.6%) on delivery. The mean neonatal birth weight was 3.1 ± 0.5 kg, with 11( 8.9%) of those categorized as low-weight neonates less than 2.5 kg. Apgar scores at 1 and 5 minutes were 7.8 ± 1.1 and 8.9 ± 0.9, respectively. As low, almost 5.7% of neonates had an Apgar score of 3 at 5 minutes. The mean gestational age at delivery for elective cesarean deliveries (CD) was 37.1 ± 2.4 weeks. Preterm deliveries, occurring between 34 and 37 weeks, were observed in 19 cases (15.4%), primarily due to elective induction or cesarean section. The overall cesarean section rate in the study was 48 cases (39%), with the most common reasons being maternal compromise due to fetal distress or repeat cesarean delivery (Table 3).

**Table 3:** Fetal outcomes

Outcome	Value
Fetal distress (%)	15(12.2%)
Oligohydramnios (%)	13(10.6%)
Meconium-stained amniotic fluid (%)	18(14.6%)
Mean birth weight (kg)	3.1 ± 0.5
Low birth weight (<2.5 kg) (%)	11(8.9%)
Apgar score at 1 minute, mean ± SD	7.8 ± 1.1
Apgar score at 5 minutes, mean ± SD	8.9 ± 0.9
Preterm delivery (%)	19(15.4%)
Cesarean section rate (%)	48(39%)
NICU admissions (%)	12(12.2%)
Meconium aspiration syndrome (%)	4(3.3%)
Stillbirth (%)	0(0%)

NICU admission was necessary in 15(12.2%) of cases, mostly for prematurity or respiratory distress. Fetal outcomes in general were reassuring, and there was no report of stillbirth. Only 12.2% of pregnancies had fetal distress and meconium aspiration syndrome was noted in only four (3.3%) neonates (Table 3).

The primary outcomes of specific interest are maternal and fetal outcomes in patients with BA levels at delivery that normalized versus those that did not normalize. Compared with those of women who had persistently elevated BA levels, the fetuses from normalized biochemical measures had a lower incidence of fetal distress 8(8.3%) vs 6(22.2%) and preterm delivery 11(11.5%) vs 7(25.9%). In addition, the rate of NICU admissions was reduced in normalized patients 7(7.3%) vs 5(18.5%) (Table 4).

**Table 4:** Comparison of outcomes between normalized and non-normalized bile acid levels at delivery

Outcome	Normalized Bile Acid Levels (n = 96)	Non-Normalized Bile Acid Levels (n = 27)	Total	p-Value
Fetal distress	8(8.3%)	6(22.2%)	14(11.4%)	0.02
Preterm delivery	11(11.5%)	7(25.9%)	18(14.6%)	0.05
NICU admissions	7(7.3%)	5(18.5%)	12(9.8%)	0.01

## Discussion

ICP is an idiopathic liver disorder unique to pregnancy, characterized by the accumulation of BAs in the liver and bloodstream, which results in pruritus and when severe, jaundice [12,13]. This condition is usually observed from the second or often third trimester. The increased maternal discomfort and higher rates of complications for the fetuses, including but not limited to preterm birth, and intrauterine fetal distress or demise, have been well demonstrated. UDCA is a naturally occurring, though relatively scarce, BA in human bile. It has been shown to effectively reduce serum BA levels and alleviate symptoms of pruritus in patients with ICP, providing significant benefits for both the mother and fetus. Over time, it has become the standard treatment for managing ICP [14].

Results of this study show that mothers noted improvements in their symptoms (especially pruritus) and that serum BA levels returned to normal after UDCA. The treatment was also linked to beneficial fetal effects, including reductions in rates of fetal distress, preterm delivery, and NICU admissions.

The most interesting finding of this study was the marked improvement in pruritus grade seen among women treated with UDCA. The mean pruritus score, a cardinal symptom of the ICP patient population, decreased from 7.3 initially to 1.8 by delivery. This improvement reinforces the results of previous clinical trials showing that UDCA decreases both BAs and pruritus in ICP patients [15,16]. Pruritus relief may, therefore, improve the general quality of life in women with ICP during pregnancy, which is arguably an important part of managing this problem.

Serum BA levels and LFTs significantly improved as a result of UDCA treatment for ICP. The fact that UDCA normalized serum BA levels in the blood of 96(78%) of the patients in our study suggests that the drug reduces the accumulation of toxic BA in the maternal blood that would otherwise lead to severe maternal and fetal complications. This finding is in alignment with the study by Nicholas [17].

However, this study appears to show a minimal rate of fetal distress and premature birth. The lowered rate of NICU admissions and fetal distress observed in women with normal BA levels suggests that UDCA may have a protective effect on the fetus. Our study observed a higher-than-expected rate of lower segment cesarean sections, with 39% of deliveries requiring surgical intervention. This elevated rate may be attributed to maternal complications such as fetal distress and repeat cesarean sections.

Improved preterm birth rates are particularly noteworthy, as ICP is well-known to be associated with early delivery. The rate of preterm delivery in this study was 18(14.6%), which is lower than the rates reported in published literature from untreated ICP populations [18,19]. This observation further underlines the promise of UDCA as a pharmacological agent that may extend pregnancy and reduce preterm delivery, something frequently burdensome in ICP management.

According to the findings of this study as well as the majority of other published research [20,21], UDCA is a safe and efficient medication for preventing the onset of ICP. Some, meanwhile, contest the advantages of UDCA therapy [22]. In a study by Feng et al., there was no proof that the administration of UDCA significantly affected neonatal death and stillbirth rates [23]. However, there were no stillbirth cases in our study since ICP was identified early in the second trimester, which allowed us to give UDCA and keep a careful eye on the patient and the fetus.

Thus, the results of this study have significant clinical value. UDCA remains indicated as a first-line medication for the management of ICP, as it should be used exclusively. Despite the evident effects on the reduction of maternal symptoms and improvement in fetal outcomes, the high variability of its effects cannot be ignored. To address the variability in UDCA's effectiveness, further research is needed to understand factors such as genetic predispositions, disease severity, and individual responses to treatment. Personalized approaches, including dosage adjustments based on patient-specific factors and more frequent monitoring, could help optimize outcomes for both mother and fetus. While UDCA remains the most effective standard therapy for managing ICP, close monitoring and individualized care remain essential.

In our study, 27 women (22%) did not achieve normalization of serum BA levels despite UDCA treatment. These cases were closely monitored, and the management approach was guided by a multidisciplinary team including maternal-fetal medicine specialists. For patients who did not respond to UDCA, dose adjustments were considered on a case-by-case basis, and additional supportive care, including regular fetal monitoring, early interventions for fetal distress, and timely delivery planning, were implemented to mitigate risks. It is worth noting that we observed a general improvement in maternal symptoms (such as pruritus) and stable liver function, suggesting that UDCA may still provide partial therapeutic benefits in these cases.

There are few limitations that should be mentioned. First, this study has an observational design and, therefore, does not allow definitively establishing causality. Randomized controlled trials are required to confirm the efficacy of UDCA and to study the optimal regime of administration. Second, the study had a relatively low number of cases, especially while analyzing the outcomes in the subgroups of patients with successfully normalized and non-normalized levels of bilateral acids.

## Conclusions

In light of its evident advantages for both the mother and the fetus, UDCA seems to be an effective ICP treatment. UDCA should be used as first-line treatment for ICP due to the benefits of improved liver function, less severe pruritus, and good fetal outcomes. Additional research is required to examine a wider range of treatment scenarios and patient responses. These results emphasize the need for tailored care in this difficult-to-treat population and broaden the body of research suggesting the potential benefits of UDCA treatment during pregnancy.

## References

[1] Cui J, Zhai Q, Chen M, Yang Z (2024). Genetically predicted lipids mediate the association between intrahepatic cholestasis of pregnancy and cardiovascular disease. Front Cardiovasc Med.

[2] Geenes V, Williamson C (2009). Intrahepatic cholestasis of pregnancy. World J Gastroenterol.

[3] Bacq Y, Sentilhes L, Reyes HB (2012). Efficacy of ursodeoxycholic acid in treating intrahepatic cholestasis of pregnancy: a meta-analysis. Gastroenterology.

[4] Williamson C, Geenes V (2014). Intrahepatic cholestasis of pregnancy. Obstet Gynecol.

[5] Misra D, Singh N, Faruqi M, Tiwari V, Kumar V, Zafar F (2024). Evaluating the utility of liver transaminases as predictors of feto-maternal outcome in lieu of serum bile acids in intrahepatic cholestasis of pregnancy: a prospective observational study. J Obstet Gynaecol India.

[6] Burrows RF, Clavisi O, Burrows E (2001). Interventions for treating cholestasis in pregnancy. Cochrane Database Syst Rev.

[7] Shemer EW, Marschall HU, Ludvigsson JF, Stephansson O (2013). Intrahepatic cholestasis of pregnancy and associated adverse pregnancy and fetal outcomes: a 12-year population-based cohort study. BJOG.

[8] Granese R, Calagna G, Alibrandi A (2023). Maternal and neonatal outcomes in intrahepatic cholestasis of pregnancy. J Clin Med.

[9] Kumari A, Kumar A, Kumar M, Swati S (2022). Feto-maternal effects of adding rifampicin to ursodeoxycholic acid in the treatment of intrahepatic cholestasis of pregnancy. Cureus.

[10] (2017). Erratum: Evaluating the effectiveness and safety of ursodeoxycholic acid in treatment of intrahepatic cholestasis of pregnancy: a meta-analysis (a PRISMA-compliant study): Erratum. Medicine (Baltimore).

[11] Avsar HA, Atlıhan U, Ata C, Erkılınc S (2024). Intrahepatic cholestasis of pregnancy and its association with preeclampsia and gestational diabetes: a retrospective analysis. Arch Gynecol Obstet.

[12] Zheng Q, Shen L, Zhao D (2021). Metabolic characteristics of plasma bile acids in patients with intrahepatic cholestasis of pregnancy-mass spectrometric study. Metabolomics.

[13] Wood AM, Livingston EG, Hughes BL, Kuller JA (2018). Intrahepatic cholestasis of pregnancy: a review of diagnosis and management. Obstet Gynecol Surv.

[14] Chappell LC, Chambers J, Dixon PH (2018). Ursodeoxycholic acid versus placebo in the treatment of women with intrahepatic cholestasis of pregnancy (ICP) to improve perinatal outcomes: protocol for a randomised controlled trial (PITCHES). Trials.

[15] Ambros-Rudolph CM, Glatz M, Trauner M, Kerl H, Müllegger RR (2007). The importance of serum bile acid level analysis and treatment with ursodeoxycholic acid in intrahepatic cholestasis of pregnancy: a case series from central Europe. Arch Dermatol.

[16] Stulic M, Culafic D, Boricic I (2019). Intrahepatic cholestasis of pregnancy: a case study of the rare onset in the first trimester. Medicina (Kaunas).

[17] Nichols AA (2005). Cholestasis of pregnancy: a review of the evidence. J Perinat Neonatal Nurs.

[18] Yeşil Y, Gündüz Ü, Dönmez A, Paşa S (2024). Evaluation of prenatal comfort, sleep, and quality of life in pregnant women with cholestasis: a cross-sectional study. Healthcare (Basel).

[19] Chappell LC, Gurung V, Seed PT, Chambers J, Williamson C, Thornton JG (2012). Ursodeoxycholic acid versus placebo, and early term delivery versus expectant management, in women with intrahepatic cholestasis of pregnancy: semifactorial randomised clinical trial. BMJ.

[20] Geenes V, Williamson C (2009). Intrahepatic cholestasis of pregnancy. World J Gastroenterol.

[21] Glantz A, Marschall HU, Mattsson LA (2004). Intrahepatic cholestasis of pregnancy: relationships between bile acid levels and fetal complication rates. Hepatology.

[22] Davies MH, da Silva RC, Jones SR, Weaver JB, Elias E (1995). Fetal mortality associated with cholestasis of pregnancy and the potential benefit of therapy with ursodeoxycholic acid. Gut.

[23] Feng F, Li J, Liao J (2024). Associations of clinical subtypes and bile acid levels of intrahepatic cholestasis of pregnancy with pregnancy outcomes. Sci Rep.

